# Supplementary material for: Proximate Composition and In Vitro Bioactive Properties of Leaf Extracts from Seven Viola Species
Source: Foods. 2025 Jan 17;14(2):302. doi: 10.3390/foods14020302 (PMC11765146; doi:10.3390/foods14020302)
Supplement: Supplementary file 1 [file foods-14-00302-s001.zip › foods-3395272-supplementary.pdf]

Supporting Information

Proximate Composition and In Vitro Bioactive Properties of Leaf Extracts from Seven *Viola* Species

Guangnian Zeng [1], Xingfan Li [2], Chunbo Zhao [1,2], Yongkang Pang [1,2], Xiongfei Luo [1,2,*] and Zhonghua Tang [1,2,*]

[1] College of Chemistry, Chemical Engineering and Resource Utilization, Northeast Forestry University,
Harbin 150040, China; zengguangnian@163.com (G.Z.); zhaochunbo1992@nefu.edu.cn (C.Z.); 2023110921@nefu.edu.cn (Y.P.)

[2] Key Laboratory of Forest Plant Ecology, Ministry of Education, Northeast Forestry University,
Harbin 150040, China; lxf1756185206@163.com

* Correspondence: lxf@nefu.edu.cn (X.L.); tangzh@nefu.edu.cn (Z.T.)

Table S1

Minerals composition of seven *Viola* leaves.

Elements (mg/kg DW)	Va	Vc	Vm	Vp	Vr	Vt	Vv
Aluminium	116.56 ± 8.62f	343.77 ± 43.09e	505.12 ± 28.48e	1676.20 ± 148.44b	821.07 ± 14.75d	2133.29 ± 92.84a	1107.06 ± 149.47c
Calcium	5611.90 ± 888.43c	19839.02 ± 2638.08a	19948.98 ± 773.24a	19203.37 ± 1785.98a	18188 ± 685.86ab	14095.94 ± 111.91b	21747.10 ± 2028.10a
Copper	4.07 ± 0.87e	9.57 ± 0.35bcd	10.52 ± 1.21bc	8.19 ± 0.16cd	17.06 ± 2.40a	11.74 ± 1.85b	6.39 ± 0.32de
Iron	118.55 ± 6.08e	333.04 ± 8.20e	655.60 ± 47.03d	1668.05 ± 43.32b	1027.47 ± 90.25c	2058.91 ± 315.24a	809.08 ± 116.01cd
Potassium	10910.83 ± 368.10d	35160.20 ± 3633.57bcd	29611.77 ± 190.43c	34094.40 ± 3442.84bc	33497.99 ± 4283.33bc	49830.38 ± 4954.19a	39571.90 ± 4562.66b
Magnesium	3302.23 ± 51.25c	7213.15 ± 511.25b	9500.45 ± 83.73a	7700.84 ± 434.25b	4702.48 ± 541.73c	4761.38 ± 659.22c	6997.71 ± 306.42b
Manganese	75.72 ± 1.40d	164.61 ± 18.46b	159.78 ± 18.6b	107.63 ± 17.66c	59.98 ± 8.18d	163.11 ± 27.03b	230.50 ± 12.66a
Molybdenum	0.10 ± 0.00d	0.62 ± 0.31cd	0.99 ± 0.01c	2.08 ± 0.88b	4.92 ± 1.57a	1.63 ± 0.51b	0.73 ± 0.22c
Sodium	126.74 ± 18.57d	354.15 ± 47.48ab	279.34 ± 1.09c	279.44 ± 5.26c	366.73 ± 14.10ab	379.26 ± 31.24a	305.85 ± 9.68bc
Zinc	75.32 ± 0.26d	156.14 ± 1.52a	92.26 ± 14.48b	88.55 ± 12.61bc	61.35 ± 0.29de	60.35 ± 10.08de	49.77 ± 8.79e

Mean ± SD values for *Viola* by different letters in the same row; significant differences ($p < 0.05$).

Table S2

In vitro antioxidant and enzyme activity inhibition capacity of seven *Viola* leaves.

Species	Va	Vc	Vm	Vp	Vr	Vt	Vv
DPPH IC_{50} (mg/L）	0.61 ± 0.07d	0.59 ± 0.05d	4.77 ± 0.36a	2.81 ± 0.09c	3.82 ± 0.17b	0.51 ± 0.08d	3.78 ± 0.49b
ABTS IC_{50} (mg/L）	0.66 ± 0.01a	0.76 ± 0.02a	0.39 ± 0.01b	0.02 ± 0.01c	0.67 ± 0.08a	0.01 ± 0.01c	0.49 ± 0.04b
FRAP (mg FE/g）	0.05 ± 0.00c	0.06 ± 0.01c	0.11 ± 0.01ab	0.09 ± 0.02b	0.11 ± 0.02ab	0.06 ± 0.01c	0.13 ± 0.02a
α-Glucosidase IC_{50} (mg/mL）	0.35 ± 0.11	2.41 ± 0.14	0.88 ± 0.36	2.02 ± 1.05	0.71 ± 0.26	2.73 ± 0.17	2.95 ± 0.21
Lipase IC_{50} (mg/mL）	0.63 ± 0.03	0.95 ± 0.02	0.88 ± 0.03	1.18 ± 0.12	0.69 ± 0.04	1.32 ± 0.07	1.07 ± 0.06

Values are expressed as mean ± SD with three replications (n = 3) for each experiment. Different letters indicate significant differences ($p < 0.05$) among treatment means as determined by Duncan's test. IC_{50} values correspond to semi-inhibitory concentrations or semi-inhibitory rates. FE: $FeSO_4$ equipment.

Table S3

The regression equation, correlation coefficient, and limit of detection of the compounds identified.

Compound	Equation of linear regression	R^2	Limit of detection (μg/ml)
p-Hydroxycinnamic acid	y = 7E+08x + 226393	0.996	5.48
Benzoic acid	y = 1E+08x + 608074	0.991	0.87
Gentianic acid	y = 2E+08x + 184280	0.991	1.52
Eugenic acid	y = 7E+08x + 82855	0.993	2.53
Cinnamic acid	y = 8E+08x + 789163	0.996	6.32
Caffeic acid	y = 9E+08x + 423086	0.995	6.79
Procatechin	y = 1E+07x + 17149	0.991	0.08
Vanillic acid	y = 4E+08x + 138454	0.990	1.45
Rosmarinic acid	y = 6E+08x + 331658	0.999	4.53
Mustelic acid	y = 8E+08x + 796467	0.993	4.16
Genistein	y = 1E+09x + 515346	0.994	5.45
Apigenin	y = 1E+09x + 523600	0.994	5.15
Naringin	y = 1E+09x + 234024	0.990	10.20
Quercetin	y = 9E+08x + 824628	0.990	10.34
Catechin	y = 5E+08x + 584145	0.991	2.01
Kaempferol	y = 6E+08x + 131996	0.995	1.99
Glycyrrhizin	y = 3E+09x + 68444	0.997	8.11
Galangin	y = 1E+09x + 234024	0.991	3.62
Rutin	y = 8E+08x + 3E+06	0.993	9.42
Genistin	y = 3E+09x + 163950	0.999	18.02
Echinacoside	y = 3E+09x + 2E+06	0.992	23.60
Isoquercitrin	y = 3E+09x + 6E+06	0.991	16.80
Esculin	y = 62.231x + 222.93	0.999	2.50
Esculetin	y = 44.883x + 449.46	0.999	40.00

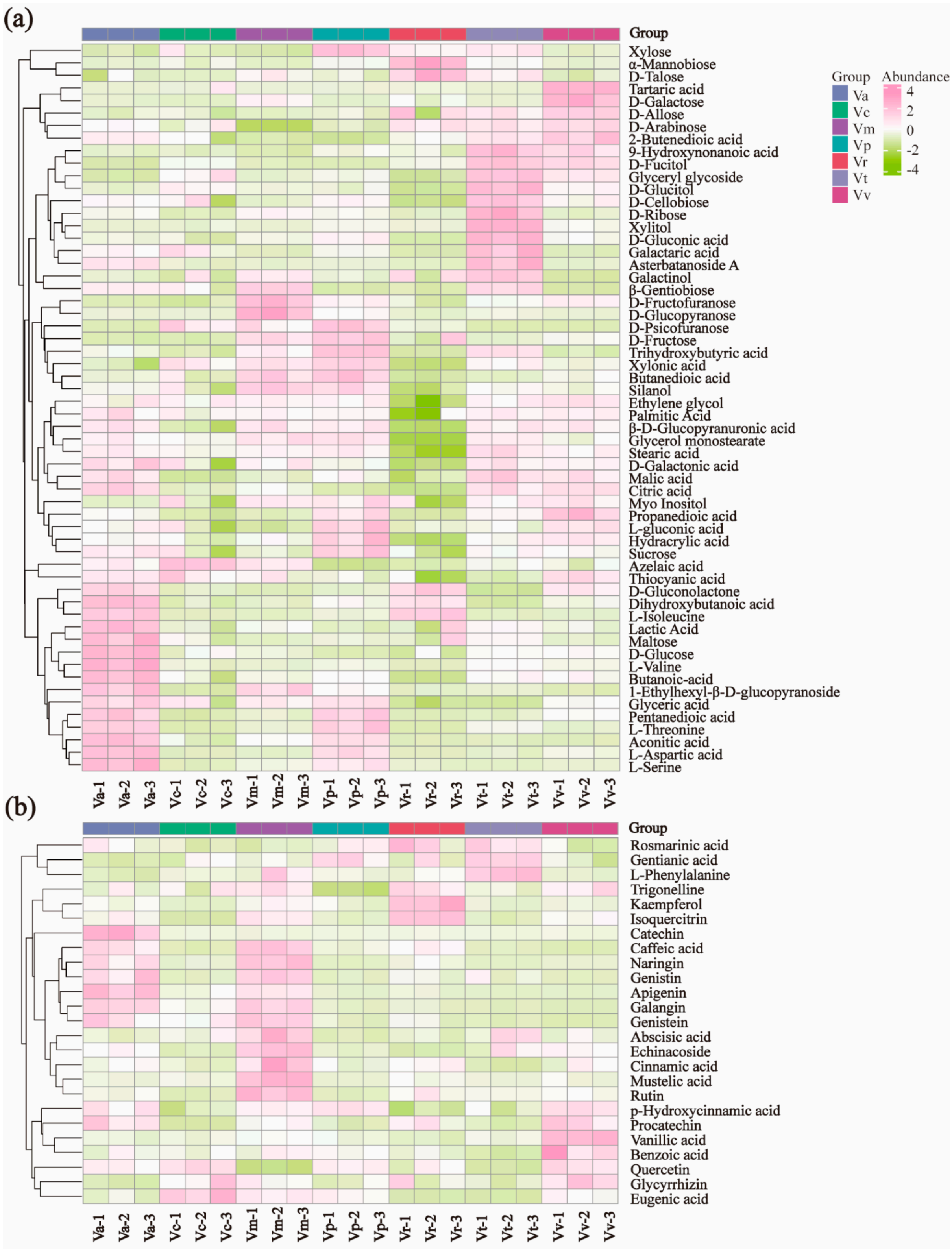

Figure S1. Heatmap illustrating the distribution of small molecules, phenolic compounds, and flavonoids in different *Viola* leaves. (a) Heatmap shows the abundance of sugars, organic acids, and amino acids, where green represents low abundance, pink indicates high abundance, and white is neutral. (b) Heatmap

details the abundance of phenolic and flavonoid compounds across the seven *Viola*

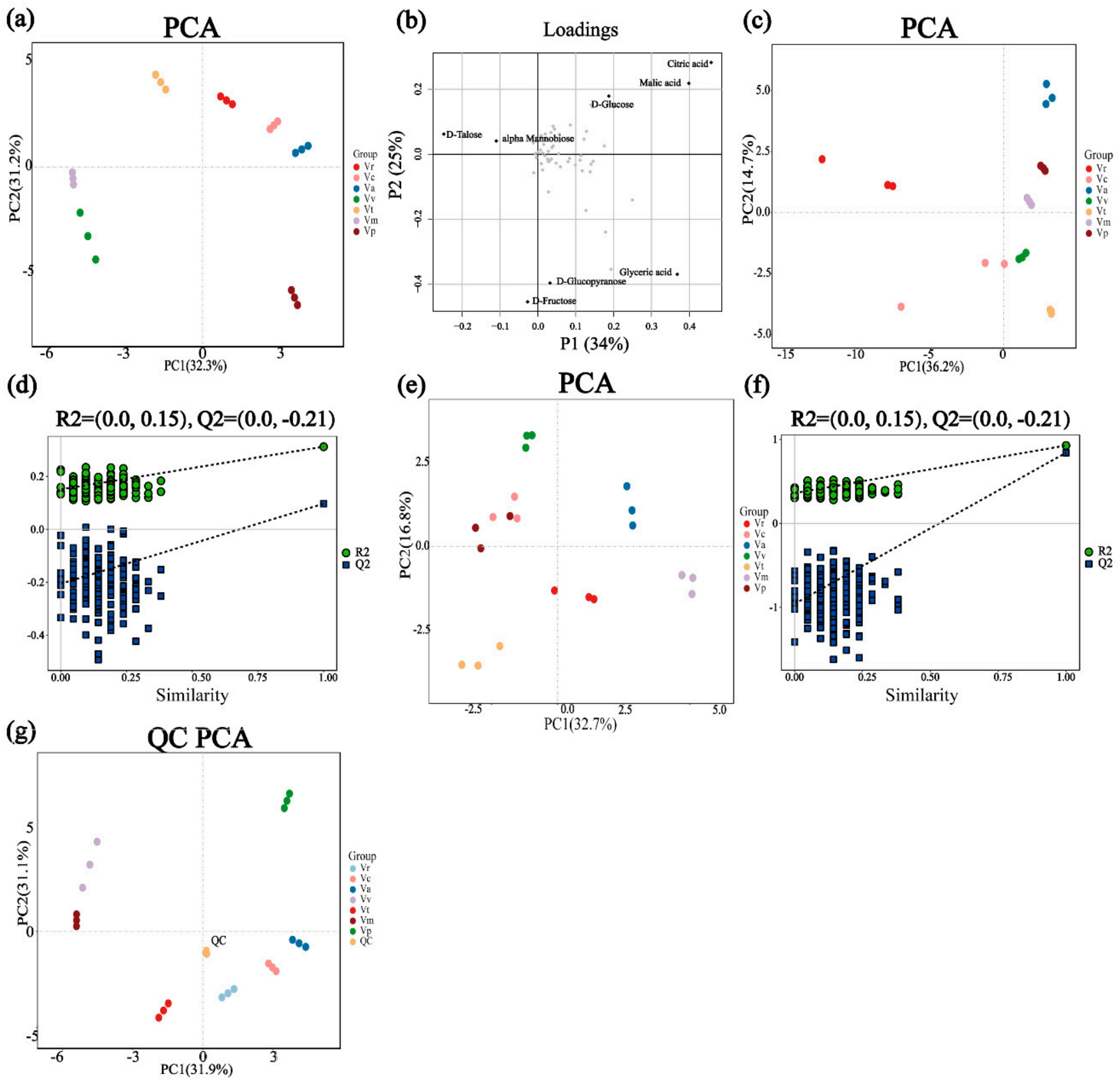

species.

Figure S2. Principal component analysis (PCA), loading plot, and permutation tests for various chemical constituents in *Viola* leaves. (a) PCA plot showing fatty acid distribution across *Viola* leaves. (b) Loading plot of small molecular compounds contributing to principal components in *Viola* composition. (c) PCA plot highlighting small molecular substances in *Viola* leaves. (d) Permutation test results for small molecular substances, displaying R^2 and Q^2 values. (e) PCA plot illustrating phenolic and flavonoid compounds. (f) Permutation test results for phenolic and flavonoid substances, with R^2 and Q^2 values. (g) Quality Control (QC) plot for fatty acid detection in seven *Viola* species.

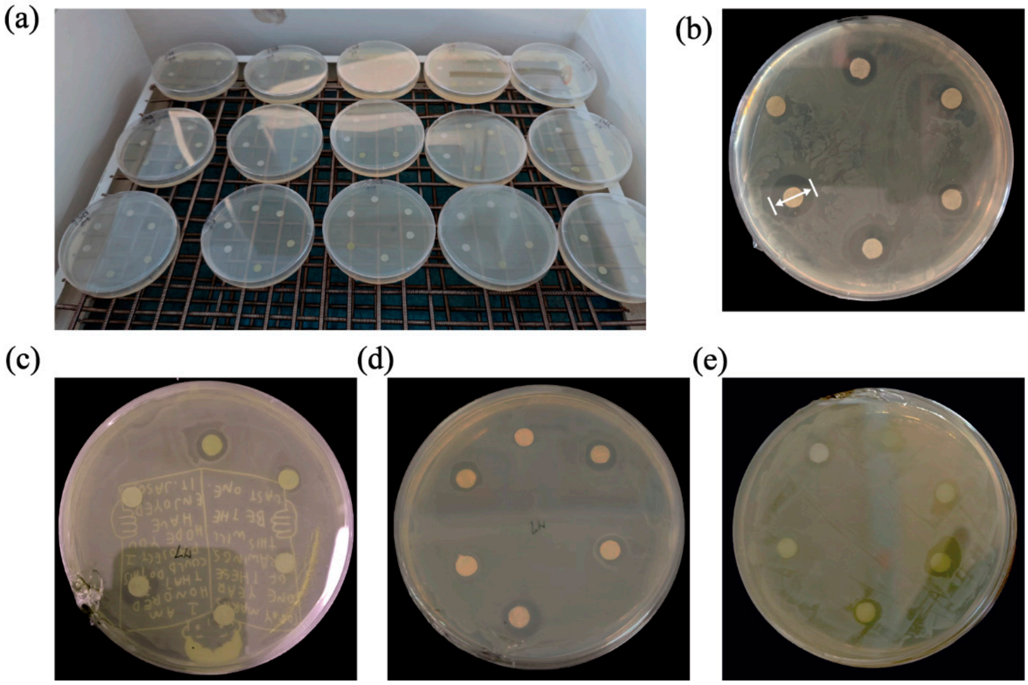

Figure **S3.** Petri dish images of antimicrobial inhibition tests for seven *Viola* species. Each dish contains one microorganism (bacteria or fungi), with six discs soaked in four different concentrations of *Viola* leaf extracts, deionized water, and kanamycin. Ethanol–water extracts of leaves from seven *Viola* species were tested against four microorganisms, with six biological replicates for each treatment. (a) Experimental image. (b) A Petri dish containing *Staphylococcus aureus*. The arrow indicates the diameter of the inhibition zone, which is used to quantitatively analyze the effectiveness of the antimicrobial agent, with its diameter reflecting the strength of the antimicrobial activity. (c) Experimental image of a Petri dish containing *Escherichia coli*. (d) A Petri dish containing *Pseudomonas aeruginosa*. (e) A Petri dish containing *Candida albicans*.

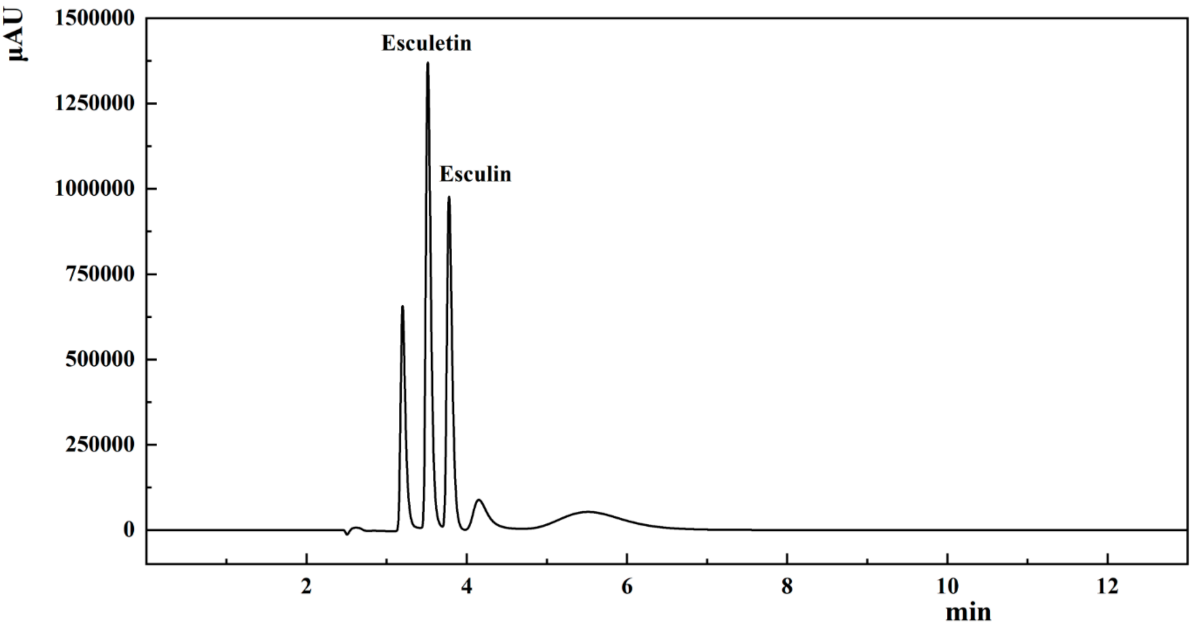

Figure S4. Chromatogram of standard compounds (compounds are UPLC-grade) with a standard concentration of 0.25 mg/mL dissolved in ethanol.

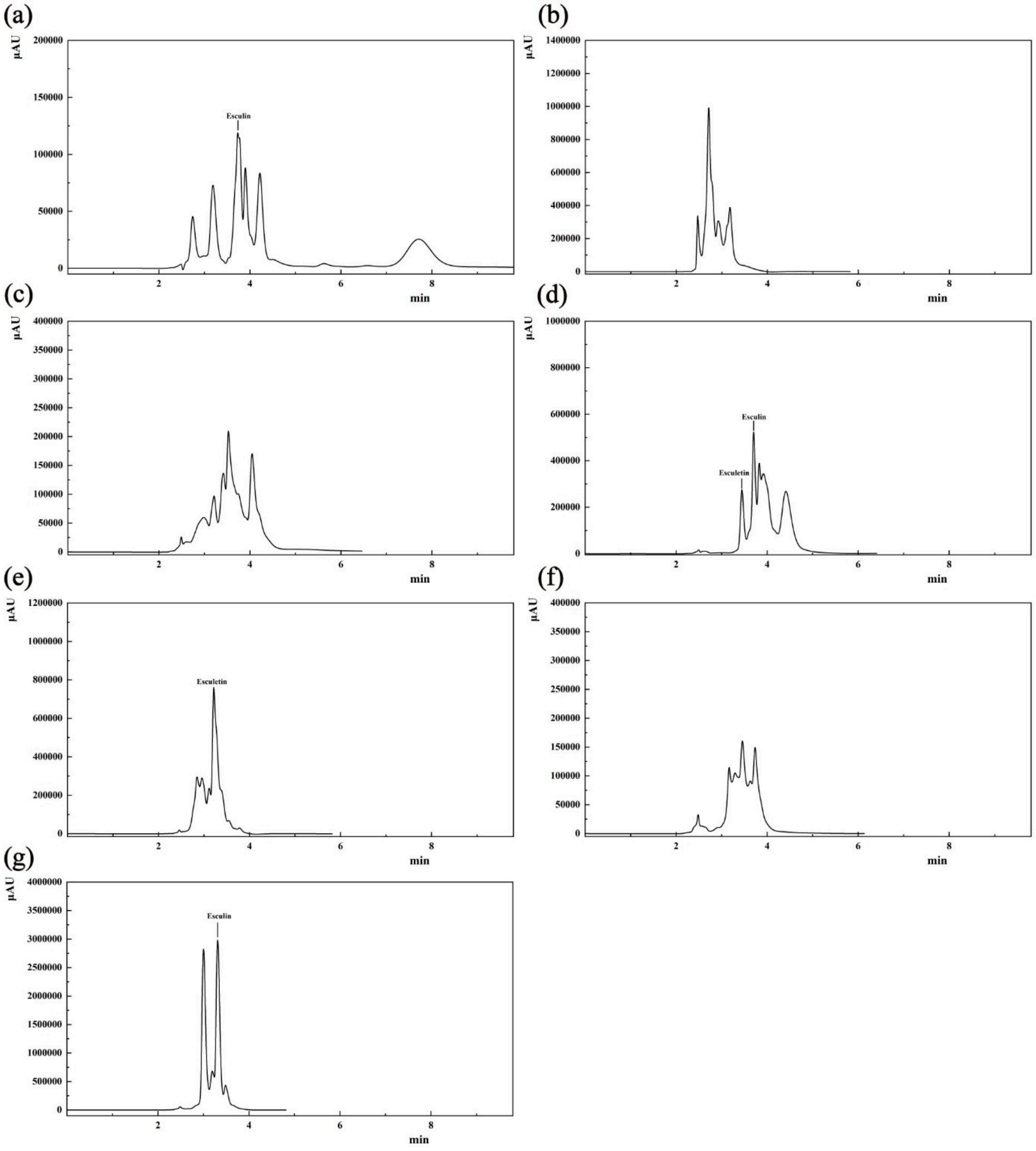

Figure S5. Chromatograms of leaf extracts from seven *Viola* species: (a) *Viola acuminata*. (b) *Viola collina*. (c) *Viola mirabilis*. (d) *Viola philippica*. (e) *Viola prionantha*. (f) *Viola tokubuchiana* var. *takedana*. (g) *Viola variegata*.
